# Breastfeeding mother’s experiences with breastfeeding counselling: a qualitative study

**DOI:** 10.1186/s13006-024-00636-x

**Published:** 2024-05-14

**Authors:** Ingvild Lande Hamnøy, Marianne Kjelsvik, Anne Bergljot Baerug, Berit Misund Dahl

**Affiliations:** 1 Norwegian University of Science and Technology, Ålesund, Norway; 2 The Norwegian Directorate of Health, Oslo, Norway; 3 University of Stavanger, Norwegian University of Science and Technology, Ålesund, Norway

**Keywords:** Mothers, Breastfeeding counselling, Experiences, Midwives, Public health nurses, Qualitative study, Hermeneutic phenomenological study, Interviews

## Abstract

**Background:**

Mothers are recommended to breastfeed their children but can find it challenging and experience breastfeeding problems. Qualified breastfeeding counselling from healthcare professionals can help mothers master breastfeeding, but there is a need to explore mothers’ lived experiences with receiving breastfeeding counselling. We aimed to reveal breastfeeding mothers’ experiences with receiving breastfeeding counselling from midwives and public health nurses (PHNs) to provide a deeper insight into the phenomenon of breastfeeding counselling, which may improve breastfeeding counselling in practice.

**Methods:**

A qualitative design with a hermeneutic phenomenological approach was used. Individual interviews of 11 breastfeeding mothers from Norway were conducted from September 2021 to 2022. Van Manen’s guided existential inquiry guided the reflective process to provide deeper insights into the phenomenon of breastfeeding counselling.

**Results:**

The study captured the meaning of breastfeeding mothers’ lived experiences with breastfeeding counselling. Three themes and eight sub-themes were found. Breastfeeding was at stake for the mothers because breastfeeding could be reduced or stopped, and qualified breastfeeding counselling from midwives and PHNs was essential for them to establish and continue breastfeeding. They needed to be perceived as both breastfeeding mothers and as women with their own needs to master everyday life during the breastfeeding period.

**Conclusions:**

This study offers insights to midwives, PHNs and others offering breastfeeding counselling by facilitating an understanding of being a breastfeeding mother receiving breastfeeding counselling. Qualified breastfeeding counselling and a trusting relationship with midwives and PHNs are essential for mothers to establish and continue breastfeeding, while deficient counselling may cause breastfeeding difficulties. Mothers need to be treated as whole and competent persons to avoid objectification and fathers/partners need to be included in breastfeeding counselling. The ‘Baby-Friendly Hospital Initiative’ should be continued, and guidelines should align with the mothers’ need to incorporate breastfeeding into their daily lives during the breastfeeding period.

## Background

Mothers often find breastfeeding more challenging than expected. However, qualified breastfeeding counselling from healthcare professionals could prevent or solve many of the breastfeeding problems they experience [1–3].

Mothers are recommended to exclusively breastfeed their infants until they are six months old and to continue breastfeeding alongside complementary food until two years or older [4]. ‘The Baby-Friendly Hospital Initiative’ (BFHI) based on the WHO/United Nations Children’s Fund’s (UNICEF) ‘Ten Steps to Successful Breastfeeding’ contains policies and procedures for how maternity and newborn services can promote, protect, and support breastfeeding and ensure that healthcare professionals provide qualified breastfeeding counselling [4, 5].

Mothers often feel unprepared to breastfeed, but report that qualified breastfeeding counselling and emotional support from healthcare professionals at the hospital and in the first weeks at home motivate them to master challenges associated with breastfeeding [6, 7].

It has been reported that to help mothers become confident in their role and strengthen the family’s experience of handling the situation, healthcare professionals should include mothers’ partners in breastfeeding counselling [8].

Mothers can be unsure of their identity as breastfeeding mothers, they may experience guilt, shame, and self-doubt during interactions with healthcare professionals [9]. Although many mothers feel their feeding choices are respected, some feel pressured to breastfeed by healthcare professionals [2, 10], while others feel pressured to introduce infant formula if the child’s weight gain does not meet expectations [10].

Mothers should be invited to a dialogue characterised by respect and support because a strong relationship with the healthcare professional marked by openness and a sense of security will enable them to seek support and help with breastfeeding when needed [10, 11], and a screening instrument can be used to assist caring dialogues about breastfeeding based on the mothers` unique breastfeeding stories [11].

Women often find that breastfeeding is a natural part of becoming a mother, providing physical and emotional rewards for themselves and the infant [12]. Nevertheless, some mothers end breastfeeding earlier than planned for different reasons, and women who strongly wish to breastfeed but cannot, need emotional support and acceptance of their grief [12, 13]. Common reasons for early weaning are perceiving insufficient milk supply and misinterpreting normal baby behaviours as milk insufficiency [14]. Healthcare professionals have a special responsibility to counsel mothers regarding understanding their babies’ behaviour and the signs of having enough milk to help them maintain milk production and feel confident to breastfeed exclusively [14, 15].

Qualified breastfeeding counselling from healthcare professionals can help mothers master breastfeeding, but there is a need to explore mothers’ lived experiences with breastfeeding counselling. Against this background, we aimed to reveal breastfeeding mothers’ experiences with receiving breastfeeding counselling from midwives and PHNs to provide a deeper insight into the phenomenon of breastfeeding counselling. This may assist in improving breastfeeding counselling in practice.

## Methods

### Design

A qualitative study with a hermeneutic phenomenological approach was used to explore mothers’ experiences with breastfeeding counselling. This design is suitable for exploring and reflecting on the meaning of human lived experiences to gain new insights into a phenomenon [16, 17].

### Setting

This study was conducted in Norway, where it is recommended that mothers breastfeed exclusively for the first six months, and subsequently together with complementary food until the baby is at least 12 months old [18]. Most children in the country are born at public hospitals [19]. After hospital discharge, the mothers receive breastfeeding counselling from midwives and PHNs working at community child health clinics [18].

The BFHI has been developed and adapted for integration into routine community child health services in communities in Norway to ensure the quality of breastfeeding counselling [20], and baby-friendly community health clinics have been associated with increases in the number of children exclusively breastfed for up to six months [3]. In addition to the public child health service, the mother-to-mother organisation Ammehjelpen provides breastfeeding information and counselling [21]. Norway offers paid parental leave for 12 months after childbirth; 15 weeks are reserved for each parent after birth, and 16 weeks can be used as the family prefers [22].

### Recruitment and sample

To gain an in-depth understanding of the phenomenon of breastfeeding counselling, mothers with rich experiences in breastfeeding were asked to participate in the study. Leaders of baby-friendly community health clinics were contacted to ask PHNs to inform mothers about the study. Inclusion criteria were mothers who had had children within the last two years, spoke Norwegian, had a child born after the 37th week of pregnancy, and were exclusively or partly breastfeeding when leaving the hospital. Mothers who were interested in participating gave their consent to the PHNs and their contact details were given to the first author. The mothers were contacted by the first author and asked if they wanted to participate, thereafter the time and place for the interviews were arranged. Eight mothers were recruited through PHNs and three via snowball sampling. Recruitment continued until the research group included enough rich descriptions of mothers’ lived experiences with breastfeeding counselling to explore the meaning of the phenomenon [16].

Eleven breastfeeding mothers from five counties and nine child health clinics participated in the study. No participants withdrew from the study. The mothers’ ages ranged from 27 to 43 years. Nine of the mothers were breastfeeding when the interviews were carried out, while two mothers had stopped breastfeeding their child at eight to nine months of age. The children’s ages varied between four and 18 months.

### Data collection

An interview guide with open-ended questions was developed by the first author in collaboration with one of the other authors [23]. The interview guide was read by two mothers with breastfeeding experience, and changes were made based on their feedback. The purpose of the questions was to gain access to the mothers’ lived experiences through concrete descriptions of the breastfeeding counselling they had received from the midwives and PHNs(Table 1).

**Table 1:** Example questions in the interview guide

Question 1	Thinking of the time you breastfed your child, how do you experience the support and help you received during this period?
Question 2	Can you describe a situation in which the midwife and PHN supported you during breastfeeding?
Question 3	Can you tell me about a situation you experienced as challenging while you were breastfeeding? *Follow-up question*: How did you feel that you were looked after by the midwife and PHN in that situation?
Question 4	Looking back on the time your child was breastfed, what do you feel are the most important experiences you gained related to counselling on breastfeeding?

Individual interviews were conducted from September 2021 to 2022 by the first author and audio-recorded. The first author strove to have an open and reflexive attitude during the interviews in line with the hermeneutic phenomenological approach [16]. Seven interviews were conducted virtually (Microsoft Teams), two were held in the mother’s home, and two in a private meeting room in a public office. The interviews lasted 45–75 min. One of the mothers sent an email with supplementary information following her interview.

### Data analysis

All interviews were audio-recorded and transcribed verbatim in their entirety by the first author. The first author and two others in the research team (BMD, MK) read the transcriptions independently several times to grasp the meaning of each text as a whole, consistent with the hermeneutic phenomenological approach [16, 17]. NVivo software was used to organise the qualitative data by naming essential phrases and paragraphs, which captured examples of the patterns related to the meaning of mothers’ lived experiences [16]. We identified preliminary themes for each interview and the preliminary themes were discussed in the research group. During further analysis, each interview was synthesised into one text, referring to the whole interview and its parts [17]. Next, the preliminary themes from all the interviews were compared and discussed in the research group before agreeing on three essential themes (see examples of the analysis in Table 2). In this process, Van Manen’s ‘guided existential inquiry’ of the five universal and interrelated lifeworld themes (existential) through which humans experience the world was used to assist the reflective process of finding the meaning of being a mother receiving breastfeeding counselling [16, 17]. An example of this included how the mother experienced the relationship with the nurse when her breasts were touched harshly.

**Table 2:** Examples of the analysis

Essential phrase	Naming	Preliminary theme	Sub-theme	Essential theme
We clarified the expectations very early, and then it was getting straight to the point with counselling (M1)	Clarify	When expectations are clarified, one becomes open to counselling	Expectations of being understood	Breastfeeding at stake
To become confident and aware of what cues my child sends, I think that has been the most important thing, that kind of competence (M6)	Own competence	Mothers need to understand their child	Treated as a competent woman	Being a breastfeeding mother and woman
What am I wondering? I wonder about everything. Breastfeeding first or last? There was no information. There is no conclusion as she said; and it is a bit annoying that there is no conclusion, but I think she must be required to give some advice then. (M4)	Lack of information	Mothers need knowledge-based advice to handle changes in the breastfeeding situation	Prepared for the next step	Mastering everyday life

### Ethical considerations

The study was conducted with the approval of Sikt (No. 784,292, 29th June 2021). All procedures were in accordance with the Declaration of Helsinki and Ethical Principles for Medical Research [24]. All participants received information about the study before the interviews and provided written informed consent. Consent forms were stored safely to protect the participants’ anonymity.

### Rigour and reflexivity

The criteria of credibility, dependability, confirmability, and transferability were used to strengthen the rigour and trustworthiness of the study [25]. All the participants were informed of the first author’s background as a PHN to gain credibility. The first author’s knowledge of the field contributed to a better understanding of the mothers’ descriptions of their experiences with breastfeeding counselling, and helped gain the mothers’ trust. Conversely, knowing the field well required a reflexive attitude to the fact that the first author’s preconceptions could influence the interview and the findings. To further strengthen credibility, quotes about the mothers’ lived experiences were presented in the study, and the themes were discussed by the research team, which consisted of three RNs, two of whom are PHNs and one is a nutritionist. The aspect of dependability was ensured by using the same interview guide in all the interviews, with only minor changes during the research process, and an awareness of being open and curious during the interview [16]. To ensure confirmability, reflexive notes were made during the research process. Using the Consolidated criteria for reporting qualitative research (COREQ) guidelines [26] ensured a careful description of the research process, which contributed to the transferability of the data.

## Results

The essential themes captured the meaning of 11 breastfeeding mothers’ experiences with breastfeeding counselling. Breastfeeding was at stake for the mothers, and qualified breastfeeding counselling from midwives and PHNs was essential for them to establish and continue breastfeeding. They needed to be perceived as both breastfeeding mothers and as women with their own needs to master breastfeeding and everyday life during the breastfeeding period. The findings are presented as three essential themes with eight sub-themes (Table 3).

**Table 3:** Essential themes and sub-themes

Essential themes							
**Breastfeeding at stake**		**Being a breastfeeding mother and woman**			**Mastering everyday life**		
*Sub-themes*							
Expectations of being understood	Confidence or despair	Broken expectations	Met as a whole person	Treated as a competent woman	Expectations of qualifications and individually adapted breastfeeding counselling	Dialogue and personal support	Prepared for the next step

### Breastfeeding at stake

Mothers had expectations that the midwives and PHNs would understand their motivation for breastfeeding. Qualified breastfeeding counselling was important to feel confident in breastfeeding, while deficient breastfeeding counselling caused breastfeeding problems and despair with a risk of breastfeeding being reduced or stopped (Table 3).

### Expectations of being understood

Mothers wished the midwives and PHNs were interested in exploring their motivation and expectations related to breastfeeding. They described that it was good to be understood by the midwives and PHNs about their choices related to the child’s nutrition and to receive counselling adapted to their needs:

We clarified the expectations very early, and then it was getting straight to the point with counselling. (M1)

Most mothers expressed high expectations for themselves to master breastfeeding. These mothers were well-prepared and motivated for breastfeeding. They wanted the midwives and PHNs to understand that they were willing to stretch far to master breastfeeding. Other mothers had little expectation that breastfeeding would work, and some decided to combine breastfeeding with infant formula or stopped breastfeeding earlier than recommended.

### Confidence or despair

Mothers described that good breastfeeding counselling helped them feel safe and confident, while a lack of quality counselling made them sad and led to a feeling of being left to fend for themselves in a new and demanding situation.

Mothers stated that it felt good when knowledgeable and competent midwives and PHNs helped them establish breastfeeding. They appreciated that the nurses took the initiative to help them, asking about breastfeeding and giving advice regarding the different aspects such as latching, pumping, and cup feeding. A mother described her experience of getting help and developing trust in the PHN:

Being taken seriously, I just felt that she was interested in helping me achieve that, and […] it was very good that it went well. Then it was so good; every time she said something, I did it and then it worked; almost became like a guru. It was like ‘oh, it actually worked’, so then you trust it even more. It became like, yes, then I do what she says. (M5)

Other mothers felt they were not met with an understanding of how challenging the first days of breastfeeding can be. Some mothers compared the process of mastering breastfeeding to taking an important exam. A mother had difficulties with breastfeeding at the start and described how difficult it was not to meet the nurses’ breastfeeding expectations. They said to her: ‘You have to try, you have to try’. Although the mother knew it was important to put her child frequently to the breast to initiate milk production, she wished the nurses had not been so reluctant to recommend infant formula and that they understood that she needed some relief in between the breastfeeding sessions.

A lack of breastfeeding counselling or poor advice in the hospital could lead to a challenging time at home. Some mothers described that the staff at the hospital were in a hurry and that they were partly left to themselves. Those mothers who experienced that the father/partner was not allowed to be at the hospital felt lonelier. It was confusing when they had to deal with many different persons providing different advice. Advice to start with bottle feeding and formula led to breastfeeding problems. If breastfeeding was interrupted for several hours because the doctor was to examine the child, it became demanding to establish breastfeeding. They felt tired, cried a lot, and did not know how to deal with the situation. A lack of information about what awaited them related to breastfeeding when they returned home made them feel little prepared and they experienced blocked milk ducts, and breast swelling without knowing how to deal with those challenges.

Owing to all these problems, mothers needed more follow-up visits to the midwives and PHNs in the child health clinic, and some of them struggled for weeks before they could breastfeed exclusively, which was their desire.

### Being a breastfeeding mother and woman

Mothers hoped that midwives and PHNs would be interested in helping and supporting them, but experienced that their expectations were unmet. Concurrently, the mothers described that they wished to be seen as whole persons and treated as competent women.

### Broken expectations

Mothers hoped that the midwives and PHNs were more engaged in their situation, and this was especially important in the first weeks after delivery when they felt vulnerable being in a new situation. The initial period after delivery was difficult owing to breastfeeding problems and deficient support. One mother experienced numerous breastfeeding difficulties but felt her problems were not taken seriously when the midwives and PHNs told her that the pain would pass and she would be fine. The PHN showed little interest in observing the mother while breastfeeding and did not investigate why it was so painful. The mother said she had to seek all the information by asking questions and did not receive helpful answers:

My midwife often replied: you can Google that. (M2)

This made her feel like the PHN did not understand her needs, and the mother felt she was not being cared for.

Experiencing a lack of support after returning home made the first weeks difficult for some mothers. A mother said that the family did not have a home visit from the midwife at the child health clinic because of a holiday. Six days after hospital discharge, they received counselling at a breastfeeding outpatient clinic in the hospital. Further, a PHN visited them several times at home during the first three weeks to weigh the child. These visits were not experienced by the mother as any qualified help to master breastfeeding, but only as confirmation that the baby’s weight gain was unsatisfactory. At the child health clinic, they met many different PHNs. Standing in a crowded waiting room in the queue among other mothers weighing their babies made the mother feel confused and dejected:

I remember standing and crying at the child health clinic and did not quite know what to do then. So it was a tiring time; it certainly was. (M8)

After three difficult weeks, they received qualified counselling with an experienced PHN in the child health clinic, and the mother managed to breastfeed.

In contrast, another mother described that she felt reassured when a PHN supported her to stop weighing so often and breastfeed on demand after the baby gained weight. She gained the courage to trust herself and breastfeed in line with the child’s needs.

### Met as a whole person

Mothers did not feel prepared for what their bodies would feel like during the breastfeeding period and were overwhelmed by breast swelling, difficulties with connecting the child to their breasts, frequent breastfeeding, and changes in their bodies. Mothers wished the midwives and PHNs would notice their need for information and advice on caring for their vulnerable bodies, but felt they were asked little about how they felt physically.

When the midwives and PHNs were most concerned about the child and how they should be breastfed, it felt like the mothers’ pain and discomfort were not taken seriously. A mother explained how the focus on breastfeeding made her feel ignored as a person with her own need for care:

Of course, they ask how you are, but there was a lot of focus on getting your baby the food. Everything else was not unimportant but maybe it could have been a little more focused on the mother too from time to time […]. You feel a bit like you’re a milk cow then […]. You’ve just given birth to a child; that’s big for me too. (M5)

During breastfeeding counselling, some mothers experienced that midwives or PHNs touched their painful breasts in a harsh way; this made them feel that their bodies had become ‘common property’. Mothers explained that they understood that this type of breastfeeding counselling was meant to help them, but it felt uncomfortable. A mother experienced another uncomfortable situation at the child health clinic, when she was asked if a PHN could observe her breastfeeding her three-month-old baby who had gained little weight. The mother felt she was mastering breastfeeding, but the situation seemed like a test, and she felt that intimate boundaries were crossed.

In contrast, some mothers felt taken care of and seen as complete individuals when the midwives and PHNs were interested in how they felt mentally and physically and asked if they had friends or family to help. A mother described a positive experience meeting a midwife who was concerned with how she experienced her body and offered to check her wounds and stitches and assured her that her body would heal.

### Treated as a competent woman

To be treated as women capable of mastering challenges and making decisions related to breastfeeding and their infant was underlined as important by the mothers. They described finding the strength to resolve difficult situations and feeling proud of mastering breastfeeding despite experiencing problems.

Mothers preferred experiences in which the dialogue with the midwives and PHNs made them feel accepted and competent. A mother described how the PHN made her feel that she could make her own choices:

The PHN has been sneaking in words like, ‘have you thought about’ or something like that to remind me a little, or nicely say, ‘now it is time for some porridge’. It is up to me to ask and decide the right thing to do. (M3)

Another mother said that she never felt pressured by the midwives and PHNs and could decide to breastfeed or stop breastfeeding, which was crucial for her to want to continue breastfeeding.

Mothers wanted to learn more about understanding their infants because they were unsure of how to interpret their child’s cues. If their baby was crying, the mothers wondered how much and how often they should breastfeed, whether their baby was hungry or full, and if they had enough milk. A mother underlined that the most important counselling she had received was gaining expertise in awareness of the baby’s cues. This made her stronger and wiser:

To become confident and aware of what cues my child sends, I think that has been the most important thing, that kind of competence. (M6)

However, mothers who had been told to breastfeed every third hour had a written breastfeeding schedule. This gave them a feeling of control over the situation, but for most, it felt demanding to use this schedule to determine when they should breastfeed instead of listening to their child and learning to understand how their body naturally regulates milk production. Mothers did not know how long they were supposed to follow these schedules, but they continued for weeks for fear they might do something wrong. This created problems with milk production and mastitis for a mother:

I sat there afterwards and was quite bitter for having been given such advice. I don’t know if I should call it hesitant advice, maybe it was right at the start, but you can’t give advice like that and then not give an end date of how long you are going to keep doing this. (M5)

Learning the natural cues of the baby gave mothers a better experience in breastfeeding and everyday life.

### Mastering everyday life

Mothers said they expected qualified and individually adapted breastfeeding counselling from midwives and PHNs to enable them to master their daily lives during the breastfeeding period. Dialogue and personal support helped them to gain trust in the midwives and PHNs and felt more confident as mothers. Knowledge-based advice adapted to the child’s development, and changes in daily life were described as important to be prepared for the next steps to come.

### Expectations of qualifications and individually adapted breastfeeding counselling

The need for information and breastfeeding counselling was described as important to overcome the various challenges they encountered during the breastfeeding period.

In the beginning, mothers wondered about normal issues related to breastfeeding, for example, how long the child should be attached to the breast or how the let-down reflex, which makes breast milk flow, works. Mothers who combined breastfeeding with infant formula requested information about helping the baby get used to a bottle, formula ingredients, and serving size. Other mothers produced more milk than needed and wanted information on achieving normal milk production.

Mothers appreciated meeting a midwife or PHN in person who could adapt the counselling to their specific needs, confirming what they were doing right and helping them resolve challenges:

Even though a lot is written about breastfeeding on the internet, what I have appreciated is that they invited me into the office, sat down, and talked about things […]. We have been lucky we have always had things explained. Yes, websites have been used; they have shown us where we can obtain information […], but we have also discussed the obtained information. It has been very nice. (M1)

In contrast, some mothers experienced meeting midwives and PHNs with limited knowledge. They described feeling frustrated if they did not get clear answers when they needed advice.

Including the father/partner in breastfeeding counselling was important for mothers. They used the word ‘we’ when speaking about breastfeeding and said that it was good to be treated like a family because they were a team doing this together. The father/partner was a support and discussion partner.

The father/partner helped by having an overview of when the child was fed, boiling bottles, looking after the siblings, and taking care of the child at night. For mothers, it was reassuring that both of them received information because it was demanding to process everything and easier to have two people remembering what was said.

### Dialogue and personal support

A personal relationship with a competent healthcare professional, which meant meeting the midwife or PHN at the child health clinic and sitting down to have a dialogue, helped mothers feel safe and prioritised. Mothers felt accepted when they could ask questions without feeling stupid and were allowed to cry and be sensitive. Mothers appreciated when the midwives and PHNs confirmed that they were doing a good job with breastfeeding their child and should not give up, conveying that they could master this together.

Mothers described that meeting kind midwives and PHNs who wanted to help and follow up gave them a feeling of being taken care of. A mother said it felt good when the PHN was supportive and able to calm her when she felt worried:

What I felt was most important for us was the ‘breastfeeding PHN’ who helped me the most and relaxed me. She didn’t focus so much on breastfeeding. She gave me specific tips about breastfeeding, but it wasn’t that we talked most about but rather that my child was strong enough. It doesn’t matter that the child doesn’t eat all day in a way; my child was robust enough. I think that’s kind of what you need to hear. […] You are very afraid that’ I don’t have enough, I’m not good enough’, but she was very supportive. What I did was good enough, and that was nice. (M8)

A mother described how she experienced quality breastfeeding counselling and developed trust in her PHN, who was competent in and worked only with breastfeeding counselling. The PHN took the time for a conversation in her office, normalised the situation, and communicated that everything was going to be fine. The PHN supported the mother in reducing bottle feeding and increasing breastfeeding, following her child’s cues instead of a feeding schedule. The mother felt confident, and as a result of their work together, the mother was finally able to breastfeed exclusively.

### Prepared for the next step

Mothers thought about the time ahead and expressed that it was important for PHNs to provide counselling concerning future changes related to combining breastfeeding with solid food.

Some mothers described meeting knowledgeable PHNs and felt well-informed about starting solid food when the child was four months old. Nevertheless, many mothers experienced a lack of knowledge among PHNs and received scant comprehensive information. Insufficient information made mothers feel frustrated and confused. A mother described how she felt when the PHN asked her about her curiosities regarding breastfeeding and solid food:

What am I wondering? I wonder about everything. Breastfeeding first or last? There was no information. There is no conclusion as she said; and it is a bit annoying that there is no conclusion, but I think she must be required to give some advice then. (M4)

Mothers explained that breastfeeding after the first six months demanded a lot from them. Nevertheless, they shared fewer experiences with breastfeeding counselling at the child health clinic after the first months and further into the second year of life, but one theme they raised was how they could combine breastfeeding with work. Long before mothers started working again, they worried about how their child would eat properly when they were not around. This led some of them to reduce breastfeeding or stop breastfeeding earlier than recommended.

## Discussion

The study of the meaning of mothers` lived experiences with receiving breastfeeding counselling revealed that breastfeeding was at stake for the mothers because breastfeeding could be reduced or stopped, and breastfeeding counselling from midwives and PHNs was essential if they should establish and continue breastfeeding. Midwives and PHNs had to consider mothers’ needs as both breastfeeding mothers and women if they should master everyday life with a breastfeeding child.

The participants strived to accommodate breastfeeding not only because they knew the positive health effects but also because it was associated with pleasure and connecting with their child, which Brown [12] highlights as an important motivation for women to breastfeed. Van Manen [16] claims that our identity is shaped by periods in life, in this case, the period of becoming a mother. Brown [12] and Yuen [13] point out that mastering breastfeeding can be associated with women’s identity, and women who wish to breastfeed but cannot may experience grief. Hence, it is significant to help women fulfil their breastfeeding wishes.

Mothers stated that they needed to learn to understand their babies’ cues and breastfeeding techniques and to deal with breastfeeding difficulties. This aligns with the findings of Pèrez-Escamilla et al. [14]., that mothers should be taught how to breastfeed after the cues of the baby because they misinterpret normal baby behaviours as milk insufficiency as a result infant formula is introduced. Research-based methods for how parents can learn about babies’ cues should be implemented in the child health services to help mothers breastfeed instead of starting with infant formula.

The results revealed that when mothers felt that midwives and PHNs had the competency to help them master breastfeeding and provided counselling in a caring manner, they developed trust in the healthcare professional and became open to receiving breastfeeding counselling. Similarly, Murphy et al. [10] and Gustafsson et al. [11] highlighted that a safe relationship and a respectful dialogue with openness for the mothers’ expectations and wishes related to the children’s nutrition is a prerequisite for helping mothers achieve their breastfeeding goals and experience care. Midwives and PHNs should facilitate mothers to tell their unique breastfeeding stories assisted by a screening instrument [11], but midwives and PHNs should also be sensitive listeners and use their clinical judgment to ensure that caring does not become instrumental as Hamnøy et al. [27] pointed out.

The findings reveal that qualified breastfeeding counselling made the mothers feel more confident in understanding their child and solving breastfeeding problems, meaning they felt empowered and enabled to master breastfeeding and manage their daily lives. This aligns with what the WHO [28] describes as the ‘empowerment process’—a health-promoting strategy.

Although many mothers had good experiences meeting midwives and PHNs, some felt isolated when they experienced a lack of support and were misunderstood by the midwives and PHNs. An example of this was when mothers felt their intimate boundaries were crossed by midwives or PHNs during breastfeeding counselling. Martinsen [29] claims that every conversation entails a risk of being rejected and violated by the other person. A person’s protective zone of untouchability can be crossed, integrity can be hurt, and the result can be closedness; consequently, the mother is not open to receiving counselling. Others found it difficult when midwives and PHNs seemed to care only about how the baby should be fed, without paying attention to their physical pain and suffering. This led to a feeling of not being seen and cared for as a whole person; one mother expressed that she felt like a ‘milk cow’ for the purpose of simply feeding the child. This aligns with Young’s [30] description of how women’s bodies and breasts tend to be objectified and their experiences related to normal reproductive processes are devalued by healthcare professionals. According to Martinsen [29], objectifying can be a threat to a healthy dialogue. This indicates that midwives and PHNs need to take the time to have a dialogue and listen to mothers to avoid objectifying them and inhibiting breastfeeding counselling and mothers’ mastery of breastfeeding. Further, mothers expressed that frequent weighing, a written schedule, or internet research disturbed their relationships with the midwives and PHNs and made them feel ignored or stressed. Counselling related to the use of objects without a dialogue seems to intensify negative emotions and foster a feeling of alienation, similar to how Young [30] describes that the use of instruments can contribute to objectification.

Some mothers experienced breastfeeding difficulties when the advice from midwives and PHNs did not follow the BFHI and WHO/UNICEF`s ‘Ten Steps to Successful Breastfeeding’ [4]. Examples of this were feeding the child every third hour and an early introduction of infant formula feeding without a medical reason. The findings underline the importance of continuing the work and implementation of the BFHI in hospitals and community health services to ensure that mothers receive qualified breastfeeding counselling, as Bærug et al. [3] and Pérez-Escamilla et al. [5] have demonstrated.

Mothers emphasised that they needed breastfeeding counselling throughout the breastfeeding period. Despite this, some mothers received little counselling after the first months, or the counselling was offered later than needed. Hence, the mothers searched for advice elsewhere to find solutions without discussing it with the PHNs. Qualified breastfeeding counselling can help mothers continue breastfeeding [1, 15], but guidelines for breastfeeding counselling and midwives and PHNs’ practice should be more aligned with mothers’ need for preparing for the challenges they will meet to promote and protect continued breastfeeding.

This study shows that mothers regard breastfeeding as a family affair and highlights that midwives and PHNs should include fathers/partners in breastfeeding counselling. Hence, to understand the phenomenon of breastfeeding counselling, fathers’/partners’ perspectives on breastfeeding counselling should be further investigated.

This study revealed limited findings concerning mothers’ experiences with breastfeeding counselling after six months and after one year. To obtain more examples of mothers’ experiences with breastfeeding counselling after the first year, more mothers with children over one year of age or with previous breastfeeding experience should be recruited. Brockway and Venturato [31] emphasised that little is written about breastfeeding after one year, possibly because mothers in Western cultures conceal that they are breastfeeding to avoid negative comments from others, including healthcare professionals. More research is needed to explore mothers’ experiences with and needs from PHNs during this latter part of the breastfeeding period. Increased knowledge about this may promote and protect breastfeeding.

### Limitations of the study

PHNs were asked to recruit mothers because they meet most of the mothers in the child health clinic, and therefore had a good overview of who had received breastfeeding counselling and met the inclusion criteria for the study. Nevertheless, this could be a limitation of the study because the mothers who were asked and accepted to participate could be those with a good relationship with the PHN and had positive experiences with the help they had received. The results show, however, that the mothers experienced both good and poor quality of the breastfeeding counselling.

Both primiparas and multiparas were included in the study to ensure the mothers had varied and rich experiences with breastfeeding counselling, which was considered a strength of the study.

Including mothers with languages other than Norwegian and from other cultures could have strengthened the study and given a broader perspective on the phenomenon of breastfeeding counselling and should be further investigated in other studies.

## Conclusions

This study provides new insight into the phenomenon of breastfeeding counselling based on a sample of mothers and their experiences as mothers receiving breastfeeding counselling from midwives and PHNs. Our study reveals that mothers consider qualified breastfeeding counselling important to help them establish and continue breastfeeding, while a lack of qualified breastfeeding counselling can lead to frustration and breastfeeding difficulties. Overall, a trusting relationship with midwives and PHNs is essential for mothers to be open to receiving breastfeeding counselling and master breastfeeding. Developing trust is dependent on midwives or PHNs’ breastfeeding competence and their ability to communicate with the family. Midwives and PHNs need to engage with mothers as unique individuals, ensuring that their experiences are addressed comprehensively, treating them as whole and competent persons rather than objectifying them. Midwives, PHNs and policymakers need to continue the ‘Baby-Friendly Hospital Initiative’ in hospitals and community health services. Guidelines should align with the mothers’ need to incorporate breastfeeding into their daily lives during the breastfeeding period.

The findings provide midwives, PHNs and others who offer breastfeeding counselling with new insights into and an understanding of being a mother receiving breastfeeding counselling which may improve breastfeeding counselling in practice.

## Data Availability

No datasets were generated or analysed during the current study.

## Abbreviations

BFHI: Baby-Friendly Hospital Initiative
COREQ: Consolidated criteria for reporting qualitative research
PHN: Public health nurse
UNICEF: United Nations Children`s Fund
WHO: World Health Organization
